# Outer membrane vesicles of carbapenem-resistant *Acinetobacter baumannii* derive an enhanced inflammatory response

**DOI:** 10.1128/spectrum.02627-25

**Published:** 2026-01-29

**Authors:** Yang Yang, Nana Tang, Yuanyuan Zeng, Jianjie Zhu, Jianjun Li, Lei Gu, Jiajia Wang, Zeyi Liu, Jian-an Huang

**Affiliations:** 1 Department of Pulmonary and Critical Care Medicine, The First Affiliated Hospital of Soochow University 12582, Suzhou, China; 2 Institute of Respiratory Diseases, Soochow University 12582, Suzhou, China; 3 Suzhou Key Laboratory for Respiratory Diseases, Suzhou, China; 4 Medical Intensive Care Unit, The Affiliated Hospital of Guizhou Medical University 74720, Guiyang, China; University of Manitoba, Winnipeg, Manitoba, Canada

**Keywords:** carbapenem-resistant *Acinetobacter baumannii*, outer membrane vesicles, inflammatory response, antibiotic resistance

## Abstract

Carbapenem-resistant *A. baumannii* (CRAB) is a critical nosocomial pathogen with limited treatment options. Although antibiotic resistance in CRAB is well-characterized, its interactions with host immunity and the contribution of outer membrane vesicles (OMVs) to pathogenesis remain poorly understood. We examined a clinical CRAB isolate and compared it with the reference strain A19606. Antimicrobial susceptibility testing revealed complete resistance of CRAB to commonly used antibiotics in clinical practice, while A19606 remained susceptible to most agents. In murine intranasal infection models and bone marrow-derived macrophages, CRAB induced significantly stronger activation of inflammatory signaling pathways and elevated levels of pro-inflammatory cytokines relative to A19606. Transcriptomic analysis of the infected lung tissue identified differentially expressed genes, enriched for inflammatory response pathways. Proteomics showed upregulated proteins in CRAB related to secretion systems. Characterization of OMVs showed that CRAB-derived vesicles were enriched in periplasmic and outer membrane proteins and elicited stronger inflammatory signaling in macrophages. CRAB displays expansive antibiotic resistance and enhanced pro-inflammatory potential mediated in part by unique OMVs properties. Targeting OMVs formation or host immune modulation may represent effective strategies for combating CRAB infections.

**IMPORTANCE:**

In this study, we comprehensively compare a clinical carbapenem-resistant *A. baumannii* (CRAB) with the reference strain A19606, examining antimicrobial resistance profiles, *in vivo* and *in vitro* immune activation, and the composition and function of extracellular vesicles (outer membrane vesicles, OMVs). We found that CRAB not only resists all tested antibiotics but also induces significantly greater activation of MAPK and NF-κB signaling and elevated cytokine production in macrophages and infected lungs. Proteomic analysis revealed that CRAB-derived OMVs are larger and enriched in proteins associated with secretion and immune activation, acting as potent pro-inflammatory mediators. These findings illuminate dual mechanisms driving CRAB pathogenicity-extensive drug resistance combined with heightened host inflammatory response-and suggest novel intervention strategies targeting OMVs-mediated immune modulation.

## INTRODUCTION

*Acinetobacter baumannii* is an increasingly prevalent and formidable pathogen in hospital environments, particularly in intensive care units and among immunocompromised or critically ill patients (1). Its remarkable ability to acquire resistance to multiple antibiotic classes, especially carbapenems, has severely limited treatment options. The rapid global spread of carbapenem-resistant *A. baumannii* (CRAB) has prompted the World Health Organization to designate it as a critical-priority pathogen requiring urgent development of new diagnostics, therapeutics, and preventive strategies (2). Infections caused by CRAB are associated with high morbidity and mortality, largely driven by therapeutic failure and the pathogen’s roles in ventilator-associated pneumonia, bacteremia, and wound infections (3, 4).

Extensive research over the last decade has elucidated multiple mechanisms driving carbapenem resistance in *A. baumannii*, including the production of OXA-type carbapenemases, metallo-β-lactamases, upregulation of efflux pumps, alterations in porins, and acquisition of mobile genetic elements (5–7). While these studies have significantly advanced our understanding of CRAB antimicrobial resistance, they provide limited insights into how CRAB interacts with the host immune system during infection. Preliminary studies suggest that CRAB infections can provoke dysregulated immune responses, including excessive cytokine release and consequent tissue damage (8, 9). However, whether CRAB strains differ from antibiotic-susceptible or reference strains in their capacity to activate innate immune signaling remains largely unresolved. Moreover, comparative analyses of immune responses elicited by clinical CRAB isolates versus laboratory reference strains under controlled experimental conditions are scarce.

Outer membrane vesicles (OMVs) have emerged as key mediators of host-pathogen interactions in gram-negative bacteria (10, 11). OMVs are naturally secreted, spherical bilayered particles that contain proteins, lipids, enzymes, toxins, DNA, and RNA and serve as vehicles for delivering effectors to host cells (12). In *A. baumannii*, OMVs have been implicated in immune stimulation, biofilm formation, horizontal gene transfer, antimicrobial resistance dissemination, and delivery of virulence factors. OMVs-associated molecules such as lipooligosaccharide (LOS), outer membrane protein A, and other immunogenic proteins can activate Toll-like receptor, MAPK, and NF-κB pathways in host cells (13–15). Nonetheless, it remains unclear whether OMVs produced by CRAB differ in abundance, size, protein cargo, or immunomodulatory activity compared to those produced by standard reference strains such as A19606. Understanding these potential differences is crucial for identifying virulence traits that may synergize with antimicrobial resistance to drive disease severity.

To address these knowledge gaps, we employed a multidisciplinary approach-including antimicrobial susceptibility testing, murine infection models, transcriptomic profiling of the lung tissue, *in vitro* macrophage assays, and proteomic characterization of bacterial cells and OMVs-to investigate immunological and molecular differences between a clinical CRAB isolate and the well-characterized reference strain *A. baumannii* A19606. This integrated strategy enables systematic comparison of bacterial features, host immune activation, and OMVs-associated properties that may contribute to CRAB pathogenicity.

It is important to note that this study focuses on a single clinical CRAB isolate. Given that CRAB strains display substantial genomic and phenotypic diversity, the immune activation patterns and OMVs-associated characteristics observed here may not be representative of all CRAB strains.

## RESULTS

### Antimicrobial susceptibility and genotyping of isolated CRAB

A clinical strain of CRAB was isolated from the sputum of a 45-year-old male neurosurgical patient. For comparison, the reference strain A19606 was obtained from the American Type Culture Collection (ATCC). Antimicrobial susceptibility testing was performed for 22 antibiotics using the broth microdilution method to determine minimum inhibitory concentrations (MICs). The clinical CRAB strain exhibited resistance to all 22 tested antibiotics, demonstrating a complete resistance profile. In contrast, the A19606 strain was resistant to only 7 antibiotics, highlighting a marked difference in susceptibility patterns between the two strains (Table 1).

**TABLE 1:** Antimicrobial susceptibility profiles of CRAB and A19606

Antibiotics	A19606		CRAB	
	MIC (μg/mL)	Susceptibility	MIC (μg/mL)	Susceptibility
Ampicillin	≥32	R	≥32	R
Amoxicillin	≥32	R	≥32	R
Ticarcillin	≤8	S	≥128	R
Piperacillin	64	I	≥128	R
Cefazolin	≥64	R	≥64	R
Cefuroxime	≥64	R	≥64	R
Cefotetan	≥64	R	≥8	R
Cefotaxime	32	I	≥64	R
Ceftazidime	16	I	≥64	R
Ceftizoxime	32	I	≥64	R
Ceftriaxone	32	I	≥64	R
Cefepime	32	R	32	R
Aztreonam	32	R	≥64	R
Dolipenem	4	I	≥8	R
Imipenem	≤0.25	S	≥16	R
Gentamicin	4	S	≥16	R
Tobramycin	≤8	S	≥16	R
Nalidixic acid	4	S	≥32	R
Ciprofloxacin	1	S	≥4	R
Levofloxacin	0.5	S	≥8	R
Moxifloxacin	0.5	S	≥8	R
Norfloxacin	8	I	≥16	R

Genotyping of the CRAB isolate was performed using average nucleotide identity (ANI) and multilocus sequence typing (MLST). MLST was conducted using the Pasteur 7-locus scheme (gltA, gyrB, gdhB, recA, cpn60, gpi, and rpoD) via the PubMLST database, which identified the isolate as sequence type ST191, one of the most prevalent CRAB STs reported in China and worldwide (16). Further genomic analysis identified the presence of multiple antimicrobial resistance genes and plasmid replicon types, suggesting that ST191 may serve as an important reservoir for the dissemination of resistance determinants.

### CRAB induces a stronger immune response than A19606

**Fig 1:**
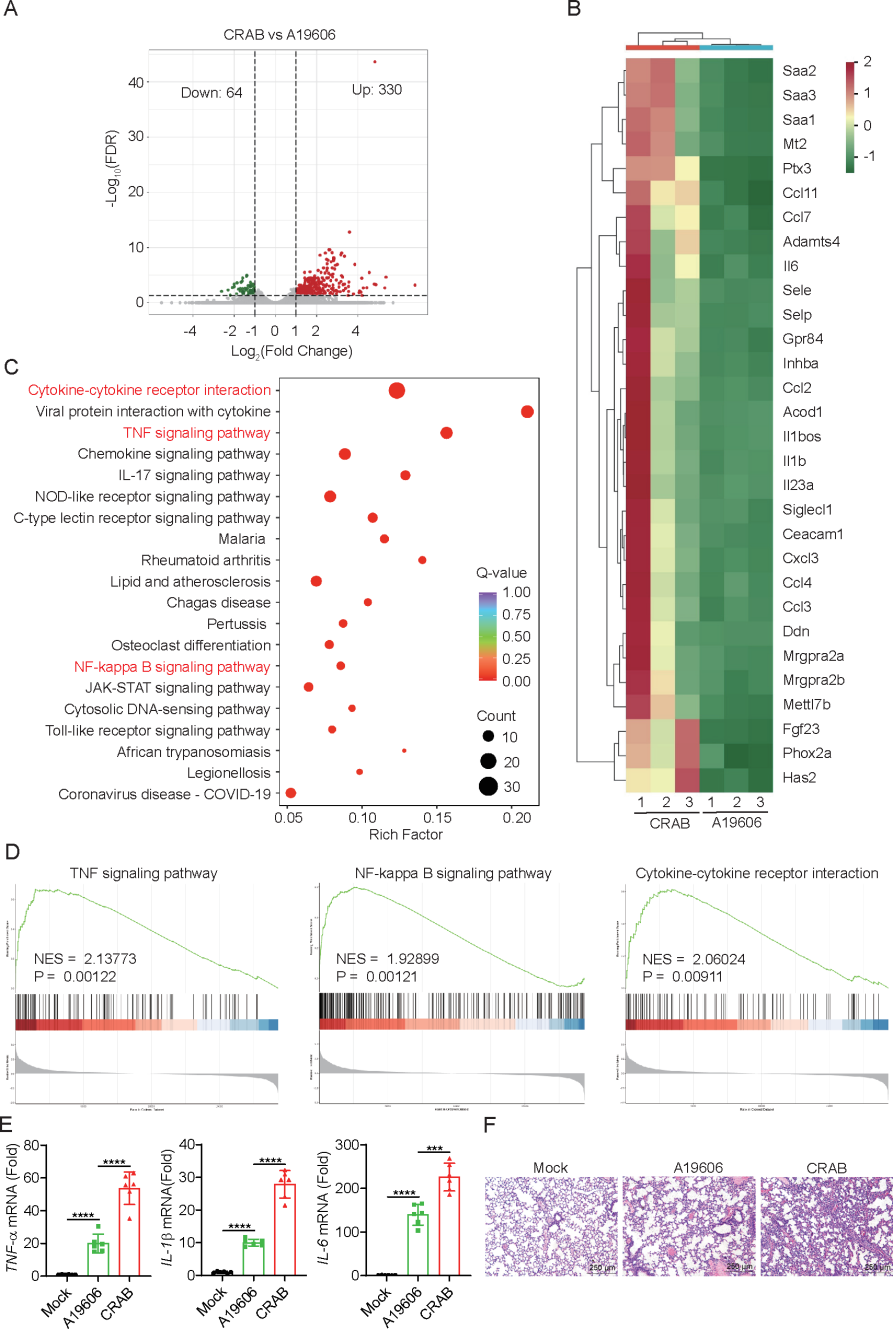
CRAB induces a stronger immune response in mice. Mice were uninfected with 1 × 10^7^ colony-forming units (CFUs) of CRAB or A19606 for 6 h (*n* = 3), and lungs were harvested and prepared for RNA-sequencing. DEGs were those with an FDR cut-off of 0.05 and a fold change of ≥±2. (**A**) Volcano plot and (**B**) heatmap showed significantly DEGs of the RNA-seq data. (**C**) Kyoto Encyclopedia of Genes and Genomes (KEGG) pathway enrichment analysis of the DEGs. (**D**) Gene Set Enrichment Analysis (GSEA) of the DEGs. (**E**) Mice were uninfected with 1 × 10^7^ CFU of CRAB or A19606 for 24 h (*n* = 6), qRT-PCR analysis of the gene expression of lung tissues (*n* = 6). (**F**) Pathological histological analysis of lung sections stained with hematoxylin and eosin (*n* = 6). Scale bar: 250 μm. The data are expressed as the mean ± SD; ***P < 0.001; ****P < 0.0001.

The host immune response plays a critical role in the pathogenesis of *A. baumannii* infections. To compare the immune responses elicited by the CRAB clinical isolate and the A19606 reference strain, mice were infected via intranasal inoculation, and lung tissues were harvested 6 h post-infection for transcriptomic analysis. RNA sequencing revealed 394 differentially expressed genes (DEGs) (≥2-fold change; false discovery rate [FDR] < 0.05) in CRAB-infected mice compared to A19606-infected controls, including 330 upregulated and 64 downregulated genes (Fig. 1A and B).

KEGG pathway enrichment analysis of the DEGs indicated significant upregulation in TNF signaling, cytokine-cytokine receptor interaction, NF-κB signaling, JAK-STAT signaling, and MAPK pathways (Fig. 1C). GSEA further confirmed enrichment in TNF signaling, NF-κB signaling, and cytokine-cytokine receptor interaction pathways (Fig. 1D). Gene Ontology (GO)-based GSEA revealed significant upregulation of genes involved in tumor necrosis factor, interleukin-1β, and interleukin-6 production (Fig. S1A and B), while Reactome-based GSEA showed enrichment in interleukin-1 family signaling, Toll-like receptor cascades, and general cytokine signaling in the immune system (Fig. S1C and D).

Consistent with these transcriptomic findings, CRAB-infected mice displayed higher pro-inflammatory expression and more severe lung tissue injury than those infected with A19606 (Fig. 1E and F). Together, these results demonstrate that the CRAB clinical isolate induces substantially stronger early inflammatory responses in the lungs compared with the reference strain.

**Fig 2:**
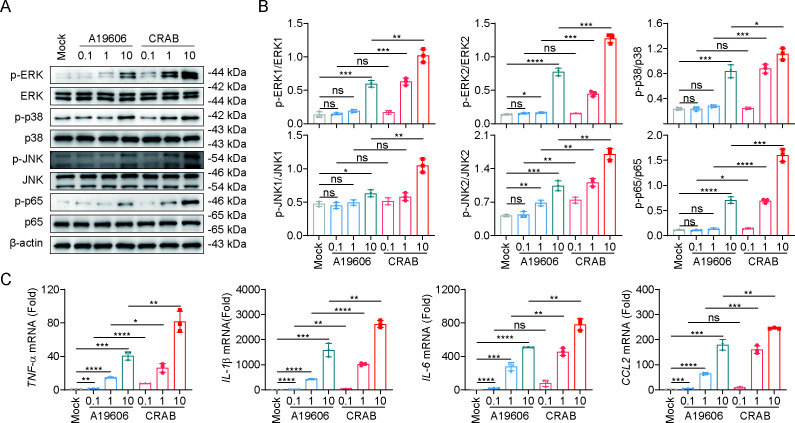
CRAB triggers a stronger immune response in macrophages. (**A**) Immunoblot analysis of protein expression in BMDMs treated with CRAB or A19606 for 2 h at a multiplicity of infection (MOI) of 0.1, 1, and 10. (**B**) Gray scale analysis summary of Western blot (WB) results from three independent experiments. (**C**) qRT-PCR analysis of gene expression in BMDMs infected with CRAB for 4 h at an MOI of 10 (*n* = 3). The data are expressed as the mean ± SD; *P < 0.05; **P < 0.01; ***P < 0.001; and ****P < 0.0001; ns, not significant.

Macrophages, as key immune cells in the alveolar space, are essential for defense against respiratory bacterial infections. To further investigate innate immune activation, bone marrow-derived macrophages (BMDMs) were infected with either CRAB or A19606. CRAB infection resulted in significantly greater phosphorylation of ERK, p38, JNK, and NF-κB p65, along with elevated expression of pro-inflammatory cytokines, compared to A19606 at equivalent infection doses (Fig. 2A and B). These data further support that CRAB elicits a stronger immune response both *in vivo* and *in vitro*.

### Proteomic analysis of CRAB reveals differential protein expression

**Fig 3:**
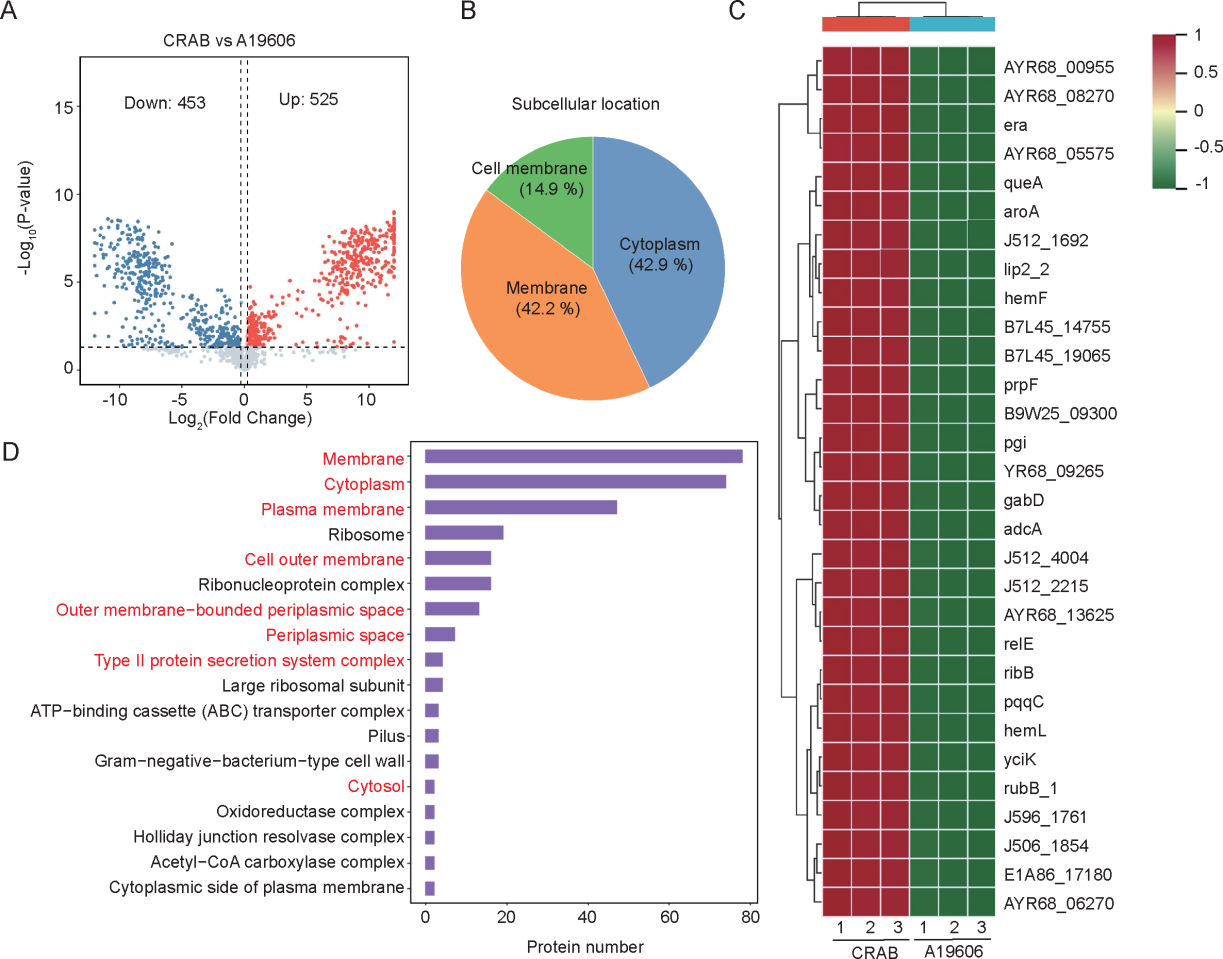
Comparison of protein expression difference between CRAB and A19606. (**A**) Volcano plot showed significantly differentially expressed proteins of CRAB and A19606 by the proteomic analysis. Differentially expressed proteins were those with an FDR cut-off of 0.05 and a fold change of ≥±1.2. (**B**) Subcellular localization analysis and (**C**) GO pathway enrichment analysis of all differentially expressed proteins. Each group had three biological replicates. (**D**) The heatmap showed top 30 highly expressed proteins in CRAB.

To explore potential mechanisms underlying the enhanced immune response induced by CRAB, we performed a comparative proteomic analysis of CRAB and A19606. The analysis identified 525 upregulated and 453 downregulated proteins in the CRAB strain relative to A19606 (Fig. 3A). Subcellular localization analysis showed that these differentially expressed proteins were distributed among the cytoplasm (42.9%), membrane (42.2%), and cell membrane (14.9%) (Fig. 3B). A heatmap depicting the top 30 highly expressed proteins in CRAB was generated (Fig. 3C).

GO enrichment analysis of all differentially expressed proteins indicated enrichment in components associated with extracellular secretion, including the outer membrane-bounded periplasmic space, cytoplasm, periplasmic space, and outer membrane (Fig. 3D), suggesting alterations in the secretion systems of CRAB compared to A19606.

### Purification and characterization of OMVs

**Fig 4:**
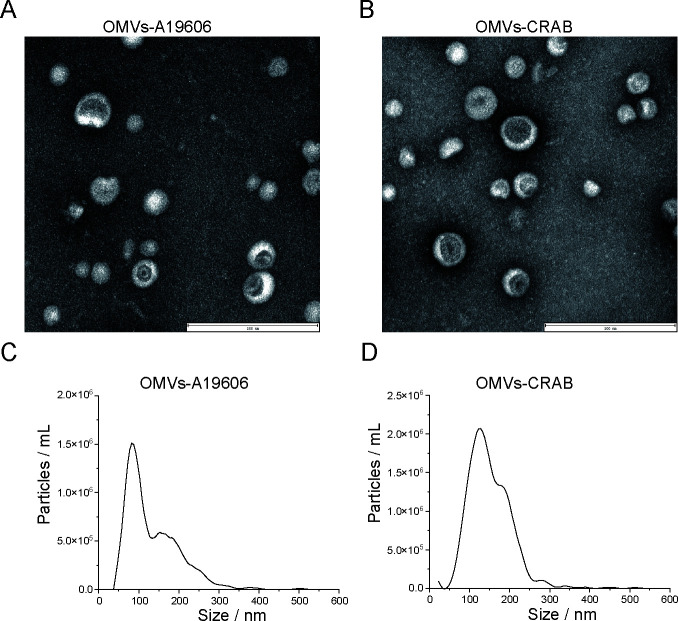
Characteristics of CRAB and A19606 derived OMVs. TEM images of OMVs derived from A19606 (**A**) and CRAB (**B**). Scale bar: 200 nm. NTA of OMVs-A19606 (**C**) and CRAB (**D**).

To determine the differences in extracellular secretion between CRAB and A19606, extracellular vesicles produced during their growth were isolated and enriched through ultracentrifugation and density gradient separation. These vesicles were designated as OMVs-CRAB and OMVs-A19606, respectively. Transmission electron microscopy (TEM) results showed that the purified extracellular vesicles possessed a distinctive spherical structure, with an intact bilayer membrane and characteristic electron density (Fig. 4A and B). Nanoparticle tracking analysis (NTA) revealed that the median diameter of A19606 extracellular vesicles was 81.6 nm, while the median diameter of CRAB extracellular vesicles was 124.9 nm (Fig. 4C and D), indicating that CRAB extracellular vesicles have a larger physical structure.

### Proteomic analysis of OMVs released by CRAB and A19606

**Fig 5:**
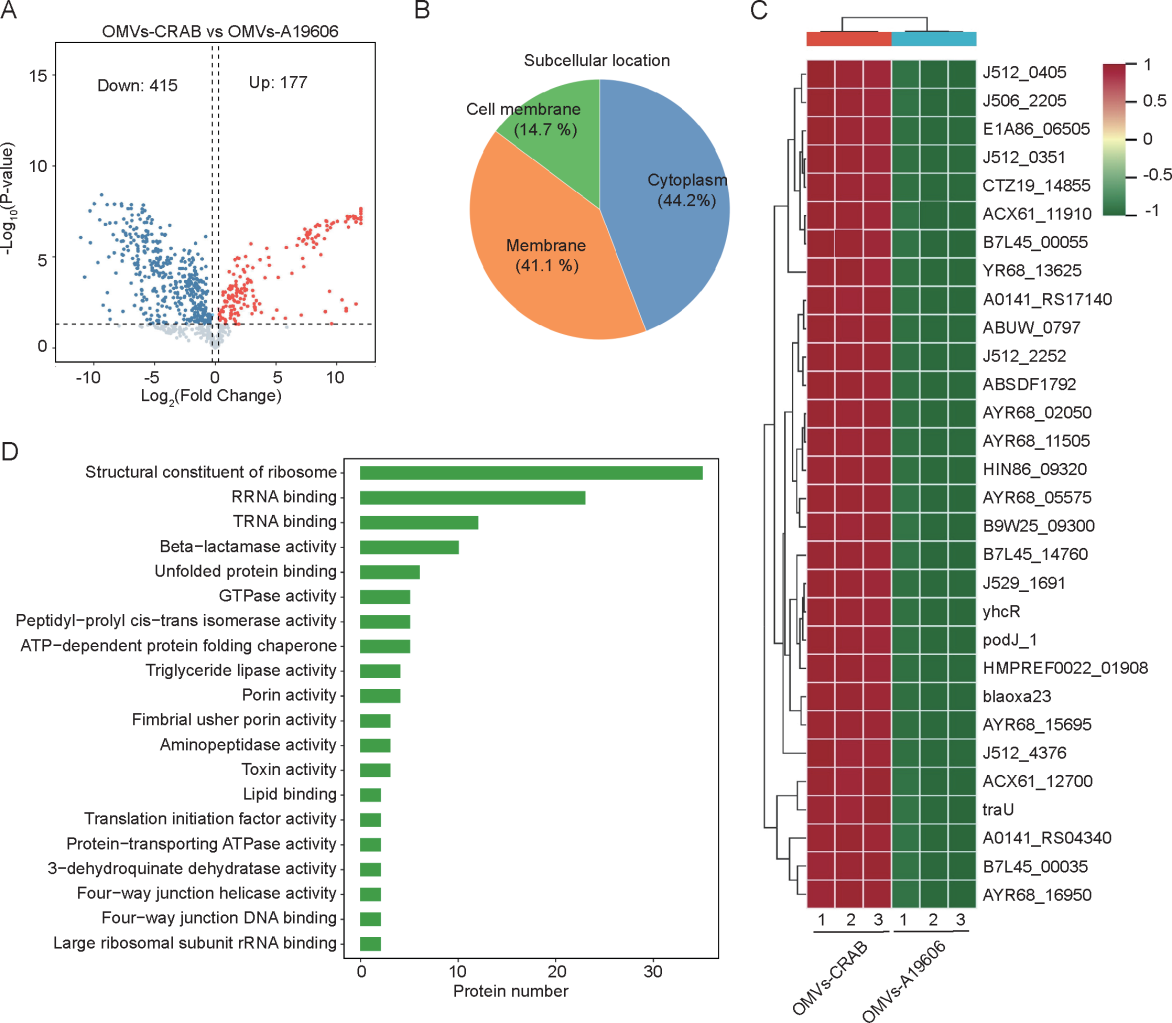
Proteomic analysis of OMVs released by CRAB and A19606. (**A**) Volcano plot showed significantly differentially expressed proteins of OMVs derived from CRAB or A19606 by the proteomic analysis. Differentially expressed proteins were those with a FDR cut-off of 0.05 and a fold change of ≥±1.2. (**B**) Subcellular localization analysis and (**C**) GO pathway enrichment analysis of all differentially expressed proteins (molecular function). (**D**) The heatmap showed top 30 highly expressed proteins in CRAB-derived OMVs.

Proteomic profiling of OMVs was conducted using LC-MS/MS following in-gel tryptic digestion. A total of 1,581 and 1,338 proteins were identified from OMVs-A19606 and OMVs-CRAB, respectively. Compared to A19606-derived OMVs, 177 proteins showed increased abundance and 415 showed decreased abundance in CRAB-derived OMVs (Fig. 5A). Subcellular localization analysis showed that differentially expressed proteins were mainly derived from the cytoplasm (44.2%), membrane (41.1%), and cell membrane (14.7%) (Fig. 5B). A heatmap was constructed to visualize the top 30 highly expressed proteins in OMVs-CRAB (Fig. 5C).

GO enrichment analysis across molecular function, biological process, and cellular component categories for all differentially expressed proteins revealed significant enrichment in terms related to structural constituents of ribosomes, β-lactamase activity, toxin-associated functions, antibiotic catabolic processes, and various membrane-and secretion-related cellular components (Fig. 5D; Fig. S2). These enrichment patterns suggest that CRAB-derived OMVs carry a diverse set of proteins associated with antibiotic resistance, stress responses, and host-pathogen interactions, further highlighting their potential contribution to CRAB pathogenicity.

### CRAB-derived OMVs trigger a stronger immune response in macrophages

**Fig 6:**
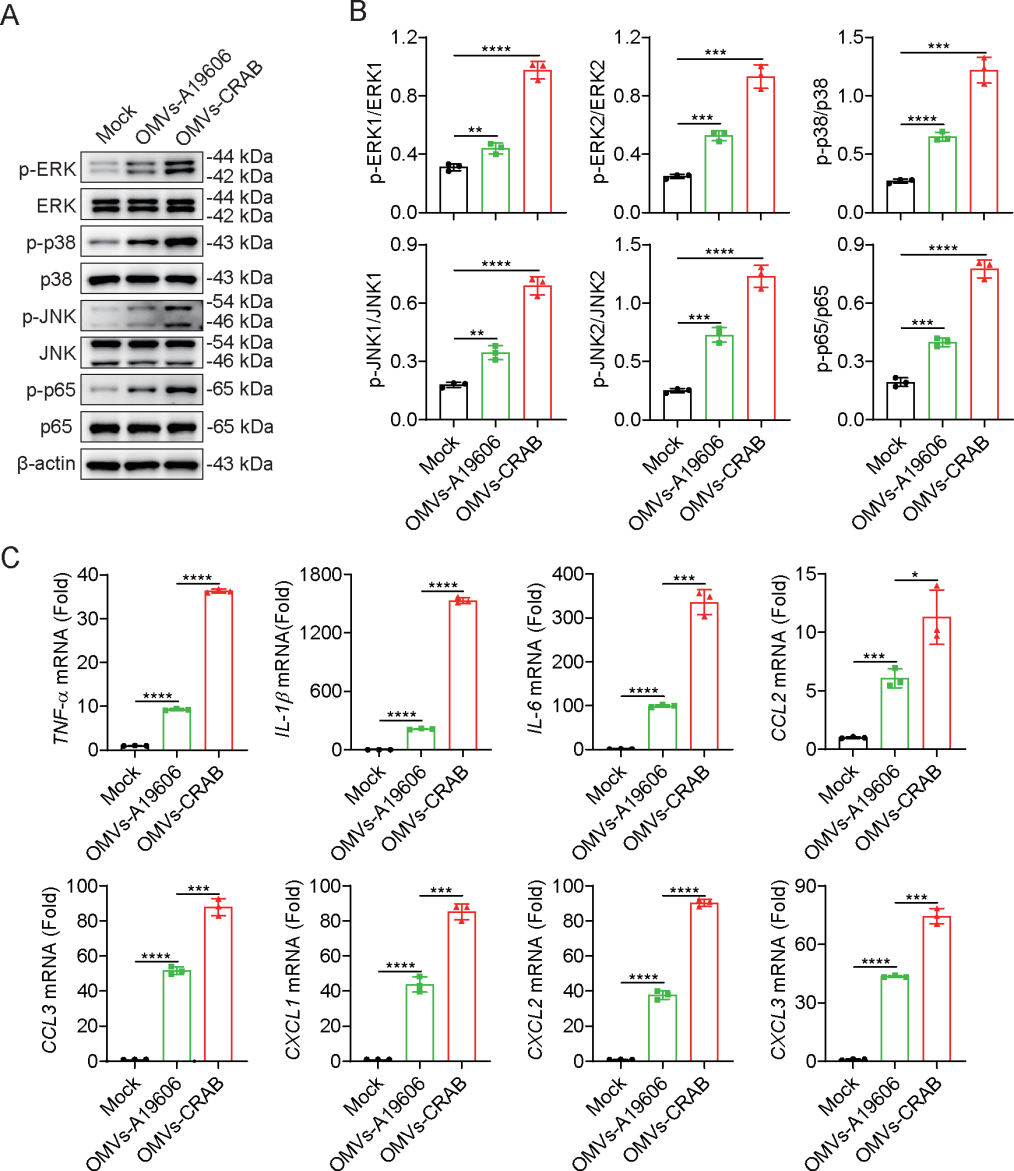
CRAB-derived OMVs trigger a stronger immune response in macrophages. (**A**) Immunoblot analysis of protein expression in BMDMs treated with OMVs-CRAB or OMVs-A19606 for 2 h at 20 μg/mL. (**B**) Gray scale analysis summary of WB results from three independent experiments. (**C**) qRT-PCR analysis of gene expression in BMDMs infected with OMVs-CRAB or OMVs-A19606 for 4 h at an MOI of 10 (*n* = 3). The data are expressed as the mean ± SD; *P < 0.05; **P < 0.01; ***P < 0.001; ****P < 0.0001.

To assess the functional implications of OMV differences, BMDMs were treated with 20 μg/mL of OMVs derived from either CRAB or A19606. OMV-CRAB treatment led to significantly greater phosphorylation of ERK, p38, JNK, and NF-κB p65, as well as elevated expression of pro-inflammatory cytokines, compared to OMVs-A19606 at the same concentration (Fig. 6A through C). These findings indicate that CRAB-derived OMVs elicit a more potent inflammatory response *in vitro*.

## DISCUSSION

CRAB has emerged as one of the most challenging nosocomial pathogens due to its rapid acquisition of antimicrobial resistance and its ability to persist in hospital environments (17, 18). While the genetic determinants of carbapenem resistance, such as OXA-type carbapenemases, efflux pumps, and porin modifications have been extensively characterized (19–21), the contribution of bacterial OMVs to pathogenesis and host immune modulation remains incompletely understood. Here, we provide a comprehensive comparative analysis of a clinical CRAB isolate and the reference strain A19606, integrating antimicrobial susceptibility testing, infection models, transcriptomics, proteomics, and OMVs characterization to propose a model in which resistance and hyperinflammatory responses are tightly linked via OMVs-mediated mechanisms.

Our results confirmed that the clinical CRAB isolate was resistant to all 22 antibiotics tested, demonstrating the extensive drug resistance of this particular strain. This extreme resistance contrasts sharply with the susceptibility of A19606, reflecting the strong selective pressures in hospital environments and the accumulation of diverse resistance determinants in clinical isolates. Recent studies have revealed that these multidrug-resistant strains not only evade antibiotics but may also possess enhanced survival and persistence traits, such as increased biofilm formation and stress tolerance (22, 23). Our findings emphasize that effective therapeutic strategies must address both antimicrobial resistance and the virulence mechanisms that underpin CRAB infections.

A key finding of our study is that CRAB induces significantly stronger immune activation than A19606, both *in vivo* and *in vitro*. In murine infection models, CRAB infection resulted in significantly elevated expression of pro-inflammatory cytokines in lung tissues. In addition, the activation of intracellular signaling pathways was examined *in vitro*, where BMDMs exposed to CRAB or its OMVs showed robust phosphorylation of MAPK pathway components (ERK, p38, and JNK) and NF-κB p65, accompanied by increased cytokine production. These observations are consistent with reports showing that *A. baumannii* can provoke hyperinflammatory responses that contribute to acute lung injury (24). Through transcriptomic profiling, we identified 394 DEGs in infected lung tissue, enriched for inflammatory signaling pathways such as TNF, JAK-STAT, MAPK, and cytokine signaling, which aligns with previous findings in other gram-negative infections (25). Our data, thus, suggest that CRAB has evolved to not only resist antibiotics but also modulate host immune signaling in a manner that may exacerbate pathogenesis.

OMVs are increasingly recognized as multifunctional virulence factors that deliver toxins, facilitate horizontal gene transfer, and shape host immune responses (26–28). We found that CRAB-derived OMVs are larger in size (median diameter ~125 nm) and have a proteome enriched in outer membrane and periplasmic proteins, particularly those associated with secretion systems. These findings echo the recent proteomic analyses of clinical *A. baumannii* OMVs, which reported enrichment of virulence-associated factors and enzymes linked to host cell damage (29, 30). Importantly, CRAB OMVs triggered markedly stronger MAPK and NF-κB activation and higher cytokine production in macrophages compared to A19606 OMVs. This suggests that the altered composition and structural characteristics of CRAB OMVs may enhance their interaction with host pattern recognition receptors, thereby amplifying inflammatory responses (31).

Our study extends prior work by combining multi-omics approaches with functional immune assays to directly compare a clinical CRAB isolate and a standard reference strain. While previous studies have individually addressed OMVs composition (32), antibiotic resistance mechanisms (33), or host-pathogen interactions (34), few have integrated these dimensions to delineate a unified pathogenic model. We propose that the enhanced immunostimulatory potential of CRAB OMVs-driven by upregulated outer membrane and secretion system proteins-represents a synergistic mechanism linking antimicrobial resistance with hyperinflammation. This dual threat may help explain why CRAB infections are associated with severe clinical outcomes, even beyond antibiotic failure. The dual role of CRAB in resisting antibiotics and promoting damaging host responses underscores the need for novel therapeutic approaches. Targeting OMVs biogenesis (e.g., via inhibition of VacJ/YrbABC complexes) or interfering with key host signaling pathways (e.g., MAPK or NF-κB) has been suggested as a viable adjunct strategy (35, 36). Our findings provide experimental support for such approaches as modulation of OMVs production or host immune signaling could attenuate both bacterial virulence and immunopathology.

Although the murine infection model used here involves immunocompetent hosts and cannot fully mimic the clinical course of CRAB pneumonia in immunocompromised patients, it remains a widely accepted and robust system for dissecting early host-pathogen interactions. This comparative framework enabled us to identify clear differences in the initial immune activation induced by the clinical CRAB isolate versus the reference strain, offering insights into strain-specific immune responses that may influence disease progression. Nevertheless, because CRAB exhibits substantial genomic and phenotypic diversity, the enhanced inflammatory activation and distinct OMVs features observed in this isolate may not be universal. Future studies involving a larger and more diverse set of clinical isolates and deeper characterization of OMVs determinants (such as LOS variants or small RNAs), as well as their potential roles in chronic infection and biofilm biology, will be essential to determine which immunological and OMVs-associated traits are conserved and to guide the development of broadly applicable therapeutic strategies.

### Conclusion

CRAB represents a dual threat, combining extensive antibiotic resistance with potent immunostimulatory capacity. By integrating antimicrobial profiling, multi-omics analyses, and infection models, we demonstrate that clinical CRAB isolates not only resist all major antibiotics but also release OMVs enriched in secretion-related and membrane-associated proteins that strongly activate MAPK and NF-κB signaling in host macrophages. These hyperinflammatory responses, coupled with the altered OMVs proteome, highlight a previously underappreciated mechanism linking bacterial resistance with host immune dysregulation. Our findings underscore the need to consider OMVs-mediated pathogenesis when developing therapeutic interventions. Targeting OMVs biogenesis or modulating host inflammatory pathways may provide a complementary strategy to conventional antibiotics. Future studies should validate these observations across diverse clinical isolates and dissect the molecular effectors within OMVs that drive immune activation and tissue injury.

## MATERIALS AND METHODS

### Mice and bacteria

C57BL/6 mice (6–8 weeks) were purchased from Shanghai Slac Animal Inc. (Shanghai, China). All mice were housed in a specific pathogen-free facility with a standard environment. All mouse experimental procedures were performed in accordance with the Institutional Animal Care and Use Committee of Soochow University, and all research protocols were approved by the Animal Ethical Committee of Soochow University. A clinical CRAB isolate was obtained from sputum cultures of a 45-year-old neurosurgical patient. The reference strain *A. baumannii* A19606 was purchased from ATCC. Strains were cultured in Luria-Bertani (LB) broth at 37°C with shaking (200 rpm) for bacterial growth and OMVs isolation.

### Animal infection

Female C57BL/6 mice (6-8 weeks) were intranasally inoculated with 1 × 10^7^ CFU of CRAB or A19606 in 50 µL phosphate-buffered saline (PBS). For RNA sequencing, after 6 h, mice were euthanized, and lung tissues were harvested for analyses.

### Whole-genome sequencing

Genomic DNA from CRAB isolates was sheared to 300-350 bp using a Covaris system, end-repaired, A-tailed, adapter-ligated, size-selected, and PCR-amplified. Libraries were sequenced on an Illumina NovaSeq 6000 (PE150). Raw reads were quality-checked with FastQC, trimmed with Trimmomatic, and assembled *de novo* using SPAdes. Assembly quality was assessed with QUAST, and genome annotation was performed with Prokka. ANI was calculated using fastANI, plasmids were identified with PlasmidFinder, and MLST typing was conducted using the Pasteur scheme via PubMLST.

### Antimicrobial susceptibility testing

The antimicrobial susceptibility of CRAB and A19606 was determined using the broth microdilution method following the Clinical and Laboratory Standards Institute (CLSI) (37). The MICs of various antibiotics against the isolated CRAB and A19606 were determined using the Vitek 2 automated system with AST-GN cards (bioMérieux, France). To ensure the accuracy of antimicrobial susceptibility results, the Kirby-Bauer disk diffusion method was employed as a supplementary test. Strains were classified as susceptible, intermediate, or resistant according to their MIC values and the interpretive breakpoints defined by the CLSI M100, 34th Edition (2024) guidelines.

### RNA sequencing

The transcriptome sequencing experiments were performed at MetWare Biotechnology (Wuhan, China). Female C57BL/6 mice (6-8 weeks) were intranasally inoculated with 1 × 10^7^ CFU of CRAB or A19606 in 50 µL PBS. After 6 h, mice were euthanized, and lung tissues were harvested for RNA sequencing analyses. Total mRNA was fragmented and reverse-transcribed into cDNA using random hexamer primers, followed by second-strand synthesis with DNA polymerase I. The resulting double-stranded cDNA was purified, end-repaired, A-tailed, and ligated to Illumina sequencing adapters. After size selection and PCR enrichment, the final cDNA libraries were sequenced on the Illumina HiSeq platform according to standard RNA-seq protocols. Reads were aligned to the Mus musculus reference genome (Ensembl GRCm39.109), and each group included three biological replicates from three individual mice. The RNA-seq data set has been deposited in the NCBI Gene Expression Omnibus (GEO) under accession number GSEA313682.

### Macrophage infection

BMDMs were differentiated from bone marrow of C57BL/6 mice in the presence of M-CSF (20 ng/mL) for 7 days. For bacterial infection, BMDMs were infected at an MOI of 0.1, 1, and 10.

### Western blot analysis

Cell lysates were separated by SDS-PAGE and transferred onto PVDF membranes (Millipore). Membranes were blocked with 5% BSA for 2 h, followed by incubation with primary antibodies at 4°C overnight. After three washes with TBST buffer (50 mM Tris, 150 mM NaCl, 0.05% Tween-20, pH 7.4), membranes were incubated with HRP-conjugated secondary antibodies for 1.5 h at room temperature. Protein bands were visualized using an enhanced chemiluminescence kit (Invitrogen) according to the manufacturer’s instructions.

### qRT-PCR analysis

Total RNA from macrophages was extracted using TRIzol reagent (A&G) following the manufacturer’s protocol, and RNA concentration and quality were assessed with a NanoDrop One spectrophotometer (Thermo Fisher Scientific). Reverse transcription was performed to synthesize cDNA, and qRT-PCR was conducted to quantify gene expression. Relative expression levels were calculated using the 2⁻ΔΔCT method.

### Isolation of OMVs

Bacterial OMVs were isolated from culture supernatants as previously described (38). Briefly, culture supernatants were filtered through a 0.22-µm sterile filter and concentrated using a 10,000 × *g* for 30 min at 4°C. The concentrates were ultracentrifuged at 150,000 × *g* for 2 h (70 Ti rotor, Beckman Coulter), and the resulting pellets were resuspended in PBS as crude OMVs. Further purification was performed using OptiPrep density gradient ultracentrifugation (5%-30% iodixanol) at 100,000 × *g* for 18 h at 4°C with a SW40 Ti rotor. The OMVs fractions were collected, washed with PBS, and subjected to centrifugation at 150,000 × *g* for 2 h at 4°C. The final OMVs suspension was filtered through a 0.22-µm membrane and plated on LB agar to confirm sterility. Purified OMVs were subsequently used for macrophage stimulation at a final concentration of 20 µg/mL.

### Proteomic analysis of bacteria and OMVs

Whole-cell and OMVs proteins were extracted using lysis buffer (4% SDS, 100 mM DTT, 100 mM Tris-HCl, pH 7.6), followed by heating, sonication, and clarification by centrifugation. Protein concentrations were determined using a BCA assay. Samples were reduced, alkylated, and digested with trypsin using a modified filter-aided sample preparation protocol. Peptides were analyzed by LC-MS/MS on an EASY-nLC 1200 system coupled to a Q Exactive HF mass spectrometer using a 120 min gradient. Raw data were searched with MaxQuant against the *A. baumannii* UniProt database. Label-free quantification was enabled, and the FDR was controlled at 1% at both peptide and protein levels. Differential protein abundance was determined using thresholds of |log2FC| ≥ 0.26 and adjusted *P* < 0.05. The proteomics data set has been deposited in the iProX with accession number IPX0014463000.

### Transmission electron microscopy

The OMVs were fixed overnight at 4°C in 2.5% glutaraldehyde containing 150 mM sodium cacodylate buffer (pH 7.4), followed by post-fixation with 1% OsO₄. Samples were stained with uranyl acetate, dehydrated through a graded ethanol series, and embedded in epoxy resin. Ultrathin sections were mounted on Formvar-coated grids, counterstained with uranyl acetate, and imaged using a Hitachi HT-7800 transmission electron microscope.

### Nanoparticle tracking analysis

Particle quantification and size profiling of OMVs were performed through NTA using a ZetaView PMX-120 system (ParticleMetrix, Germany). Particle concentration was calculated using integrated NTA software version 2.3.

### Statistical analysis

Data are presented as mean ± SD from at least three independent experiments. Statistical comparisons were made using two-tailed Student’s *t*-test or ANOVA with *post hoc* tests in GraphPad Prism. A *P*-value < 0.05 was considered statistically significant. Antimicrobial susceptibility testing was interpreted according to CLSI breakpoints and was not subjected to statistical analysis.

## Data Availability

The RNA-seq data have been deposited in the NCBI Gene Expression Omnibus (GEO) under accession number GSE313682. The mass spectrometry proteomics data have been deposited to the ProteomeXchange Consortium (https://proteomecentral.proteomexchange.org) via the iProX partner repository (39, 40) with the dataset identifier PXD073433.

## SUPPLEMENTAL MATERIAL

Figure S1 spectrum.02627-25-s0001.tif CRAB induces a stronger immune response in mice. (A) GO pathway enrichment analysis of the differentially expressed genes. (B) GO-based GSEA of the differentially expressed genes. (C) Reactome pathway enrichment analysis of the differentially expressed genes. (D) Reactome-based GSEA of the differentially expressed genes.

Figure S2 spectrum.02627-25-s0002.tif Proteomic analysis of OMVs released by CRAB and A19606. (A) GO enrichment analysis of differentially expressed proteins (biological process). (B) GO enrichment analysis of differentially expressed proteins (cellular component).

ASM does not own the copyrights to Supplemental Material that may be linked to, or accessed through, an article. The authors have granted ASM a non-exclusive, world-wide license to publish the Supplemental Material files. Please contact the corresponding author directly for reuse.
