# Supplementary figures and images for: Outer membrane vesicles of carbapenem-resistant Acinetobacter baumannii derive an enhanced inflammatory response
Source: Microbiol Spectr. 2026 Jan 29;14(3):e02627-25. doi: 10.1128/spectrum.02627-25 (PMC12955412; doi:10.1128/spectrum.02627-25)

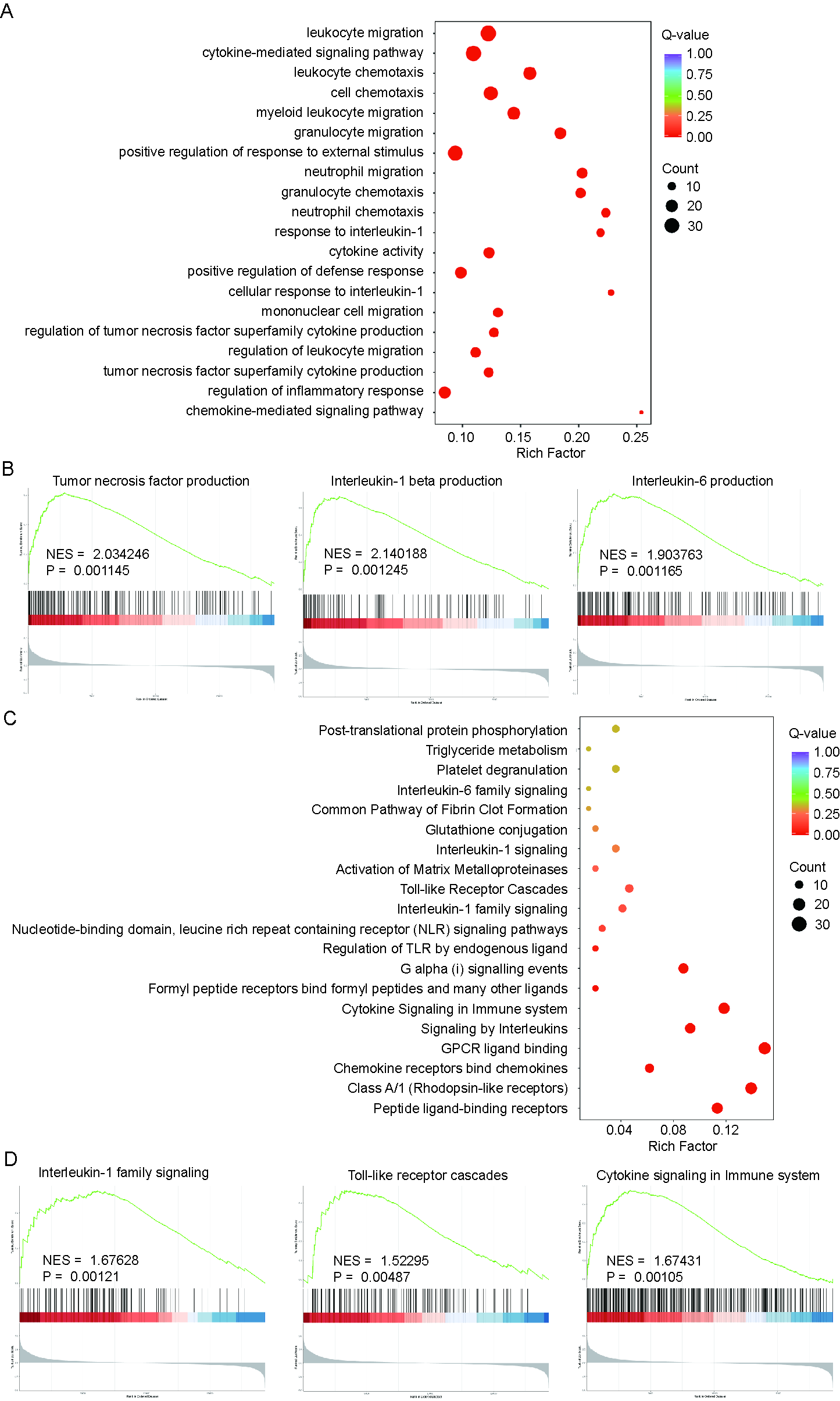

Supplement: Figure S1 — CRAB induces a stronger immune response in mice. (A) GO pathway enrichment analysis of the differentially expressed genes. (B) GO-based GSEA of the differentially expressed genes. (C) Reactome pathway enrichment analysis of the differentially expressed genes. (D) Reactome-based GSEA of the differentially expressed genes. [file spectrum.02627-25-s0001.tif]

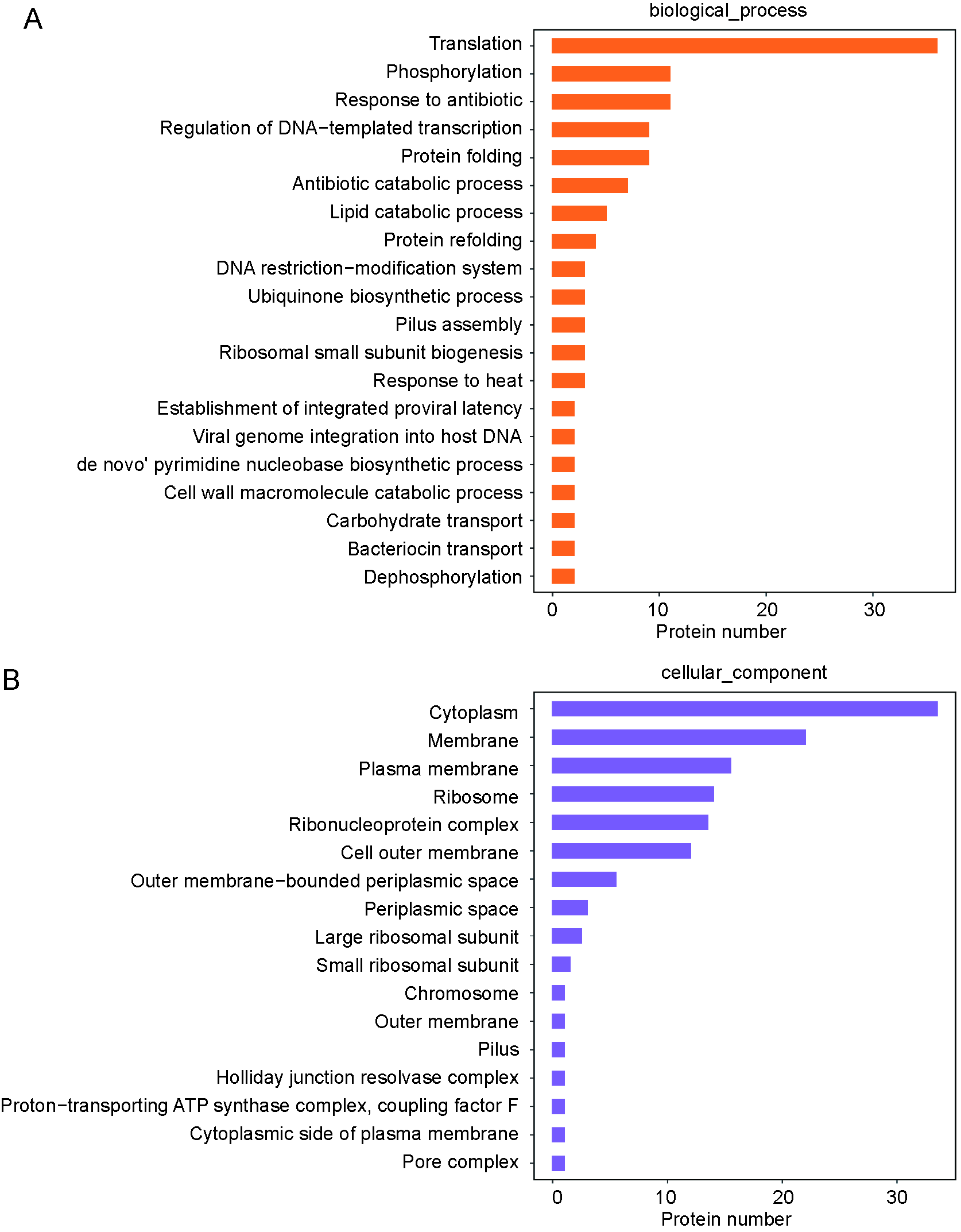

Supplement: Figure S2 — Proteomic analysis of OMVs released by CRAB and A19606. (A) GO enrichment analysis of differentially expressed proteins (biological process). (B) GO enrichment analysis of differentially expressed proteins (cellular component). [file spectrum.02627-25-s0002.tif]
